# Seasonal weather conditions affect training program efficiency and physical performance among special forces trainees: A long-term follow-up study

**DOI:** 10.1371/journal.pone.0206088

**Published:** 2018-10-18

**Authors:** Wissem Dhahbi, Maha Sellami, Anis Chaouachi, Johnny Padulo, Mirjana Milic, Imed Mekki, Karim Chamari

**Affiliations:** 1 Tunisian Research Laboratory “Sport Performance Optimisation”, National Center of Medicine and Science in Sports (CNMSS), Tunis, Tunisia; 2 Qatar Police College, Training Department, Doha, Qatar; 3 Tunisian National Guard Commandos School, Oued Zarga, Tunisia; 4 University of Qatar, College of Arts and Sciences (Qu-CAS), Sport Science Program (SSP), Doha, Qatar; 5 University eCampus, Novedrate, Italy; 6 University of Split, Faculty of Kinesiology, Split, Croatia; 7 Athlete Health and Performance Research Centre, ASPETAR, Qatar Orthopaedic and Sports Medicine Hospital, Doha, Qatar; Nottingham Trent University, UNITED KINGDOM

## Abstract

The purpose of the present investigation was to follow-up the effect of specific commandos’ training-cycles (SCTCs) on upper-body strength resistance and running endurance performance, as well as determine whether variation in seasonal parameters has any effect on physical performance. Fourteen SCTCs were held over eight years, involving 466 participants. Participants were assigned to four subgroups according to their distribution over the seasons: summer (*n* = 124), autumn (*n* = 145), winter (*n* = 52) and spring (*n* = 145). Before and after each SCTC, four tests (maximal pull-up, push-up and sit-up repetitions in 70-seconds for muscle strength resistance) and a 5-km cross-country run (endurance) were performed. Seasonal data were continuously recorded during all SCTCs. Body mass decreased significantly (*p*<0.05) in all groups following SCTCs. These training-cycles induced a significant increase (*p*<0.05) in the 70-seconds push-ups, pull-ups and sit-ups and a decrease (*p*<0.01) in the 5-km cross-country running time among all trainees. The main effect of the season was present in all tests (*p*<0.01). With regard to the percentage of changes, the results from the 70-seconds push-up, pull-up and sit-up tests were significantly higher in winter and spring (*p*<0.01) compared with the two other seasons, while 5-km cross-country performance improvements were significantly higher (*p*<0.01) in spring and summer, compared to the two other seasons. In summary,14-week of SCTCs improved upper-body strength resistance and running endurance performance in the commandos. Improvements in strength resistance performance were greater during cool weather (winter and spring), while improvements in running endurance performance were higher during hotter (spring and summer) seasons.

## Introduction

“Commandos” are special forces soldiers (SFSs) trained to conduct special/dangerous operations (i.e., foreign internal defense, offensive actions, hostage rescues and counterterrorism missions) using special technical and tactical methods [1]. A commando recruit should be able to sustain hard environmental and psychosocial challenges, which demand high levels of physical fitness [2, 3]. In terms of special forces, fitness involves high endurance and strength performance, fatigue resistance, motivation and survivability [2, 3]. As such, a specific training program based on endurance training has been developed for commandos in several countries [4]. However, the inclusion of strength/resistance training has been neglected in these programs, which is why there is the need to develop new programs that include both endurance and strength training for SFSs [5].

The goal of special forces training is to reach or maintain the physical performance level required for more demanding occupational requirements or deployment standards. In sports training, total training load, nutrition and recovery are typically planned in an individual way in order to optimize training adaptations and minimize training-related injuries and overtraining. All SFSs receive the same program and experience the same conditions in their training [6], which is more demanding than those for elite athletes. It includes strenuous physical activity and periods without sleeping [7, 8]. Such training generates high levels of stress with the aim of simulating combat conditions [9], with candidates kept physically active for 16–22 h a day [7, 10].

Military training and operations consist of tasks that can be completed through combined strength and endurance training [11]. The analysis of the components of fitness involved in the physically demanding tasks that are carried out by rangers in battle has shown that ranger regiments have a high standard of fitness abilities and fitness programs, which are mainly focused on aerobic endurance [12]. On the other hand, Vickers and Hodgdon [13]concluded that fitness programs for urban operations should focus on muscle strength of the upper and lower body and high levels of power, suggesting that an obstacle polygon (i.e., training method based on series of obstacles; uphill running, throwing grenades, shooting, lifting and carrying and running, to cross it against time [7]) could be used to develop the required stamina and strength. Many studies have confirmed that a combination of strength and endurance training is necessary for the performance of specific military tasks, such as walking with a backpack load [7, 14], and that upper-body strength and muscular development are perhaps the most important parameters. Therefore, it can be concluded that combined strength and endurance training is the foundation of soldiers’ physical performance.

Numerous factors, such as age, genetics, diet, social and psychological variables, and fitness level, have been reported to influence endurance capacity and strengthen the endurance of well-trained athletes. Several studies have demonstrated a strong link between these factors and external temperature and humidity [15]. However, changes in physical performance under a range of weather conditions are still poorly discussed.

In hot environmental temperature, the challenge of maintaining heat balance is much greater. The physiological changes during exercise in a hot environment include an increase in skin blood flow, higher heart rate to support metabolism [15], vasodilation in skin blood vessels to dissipate the heat from the skin and activation of sweat glands and sweating [16, 17]. According to Johnson [18], they suggested that combined thermoregulation and exercise under high temperature resulted first to cutaneous vasocontrition followed by vasodilation as exercise continues under the heat-stress condition.

It appears that an internal temperature, particularly that of the higher upper-nerve centers, close to 40°C is also the origin of early fatigue, as confirmed by the results of electroencephalography [19]. Hence when it comes to exercise training in highly trained soldiers, fitness staff and chain of command need to take external temperature into account. Given their lifestyle (wide range of locations and climates), SFSs are the most affected by these environment-related effects on muscle performance. SFSs are therefore forced to perform high training volumes in order to withstand the demands of the respective physical challenges in different climates. Hence, training in a cold environment should increase endurance and tolerance to training load over time [20]. However, for high-intensity exercises, such as jumping, sprinting or lifting weights, it has clearly been established that the warmer a muscle is, the more it is able to produce force in a very short period of time [20]. According to Bottinelli et al. [21], optimal muscle force is temperature-dependent and achieved in a range of 12–17°C, but less dependent between 17°C and 22°C. In addition, humidity, wind and radiant heat represent other determinants of environmental conditions that may affect muscle performance, which in turn should be taken into consideration when training and/or carrying out special tasks [22]. For example, Hayes et al. [22] observed a significant increase in oxidative stress in a trained group of soldiers (United States Marines) during a 24-day training period in a cold and humid climate.

At the time of writing, there are no studies available that have compared and quantified the effects of weather conditions on physical performance during SFS training. Therefore, the aim of this study was to (i) examine the effects of specific commandos’ training-cycles (SCTCs) which included physical fitness, technical and tactical training courses, on physical performance of SFSs and (ii) determine whether SCTCs gains could be affected by seasonal variations in weather conditions.

## Materials and methods

Participants reviewed and signed consent forms approved by the local Ethics Committee for Human Research (ECHR) of the National Guard Commandos School committee of Tunisia: “Commandos scientific staff” composed of officers and experts and in accordance with the ethical standards of the 1964 Helsinki Declaration. The study protocol described in this manuscript was reviewed and approved by this committee and all participants were volunteered and could decline participation in this research with no consequences for their status in the training program.

### Experimental approach to the problem

A longitudinal repeated cross-sectional study design was used, involving repeated observations over an eight-year period (from March 2007 to May 2015). This study investigated the effects of an SCTC program on physical performances of commando soldiers, and its variations across the four seasons of the year. The program, which consisted of 14 training-cycles each lasting 14 weeks, ran over eight years and included 466 participants. The training-cycles were grouped into four subgroups (summer, autumn, winter and spring) according to their distribution over time, with a tolerance of 2 weeks. Participants were also assigned to four subgroups according to the seasons: summer (*n* = 124), autumn (*n* = 145), winter (*n* = 52) and spring (*n* = 145). It should be noted that four out of the 17 training-cycles of this study were removed, as they took place in-between two seasons.

Before (P1) and after (P2) the experimental period, all participants completed a testing protocol, including endurance and body-weight resistance exercises, with four tests in total: maximum standard repetitions over 70-seconds for push-ups, pull-ups and sit-ups, and 5-km cross-country tests. Tests were performed with 20 min recovery between tests. However, between-days reliability and sensitivity of each test was assessed.

### Participants

For this study, 466healthy, trained antiterrorism male soldiers (Tunisian National Guard commandos) volunteered to participate to this study. All participants submitted to a health inspection before the testing, and only those with an adequate health status and doctor’s permission were allowed to participate in the study. Inclusion criteria included no contraindications to maximal exercise testing, such as cardiovascular or pulmonary risk conditions, no history of chronic disease, illness, surgeries, hospitalizations, and musculoskeletal or joint injuries during the last six months before the beginning of the experiment. Eligible participants (age: 24.17±2.48 years, body mass: 76.75±6.74 kg, body height: 178.26±5.34 cm, and BMI: 24.16±2.06 kg·m^-2^) were distributed into 13 training-cycles as described in Table 1.

**Table 1:** Participants and Training-Cycles characteristics and its chronological-seasonal distribution.

	Training-Cycles	Participants (N = 466)	Conditions
Summer N = 124	2009	N = 29 Age (years) = 24.93±2.10 Body mass (kg) = 77.62±7.28 Height (cm) = 179.52±4.72 BMI (kg·m^-2^) = 24.06±1.70	Temperature (°C) = 31.00±2.65 Humidity (%) = 67.33±12.91 Barometric pressure (hPa) = 1014.67±2.31 Visibility (km) = 11.70±0.66 Wind speed (km·h^-1^) = 10.00±2.65
	2010	N = 16 Age (years) = 24.56±1.90 Body mass (kg) = 81.25±5.80 Height (cm) = 179.69±4.54 BMI (kg·m^-2^) = 25.14±1.06	Temperature (°C) = 25.33±1.53 Humidity (%) = 58.00±11.63 Barometric pressure (hPa) = 1013.33±2.52 Visibility (km) = 9.17±1.53 Wind speed (km·h^-1^) = 7.00±2.65
	2013	N = 35 Age (years) = 26.09±1.99 Body mass (kg) = 78.31±7.70 Height (cm) = 178.83±4.84 BMI (kg·m^-2^) = 24.44±1.55	Temperature (°C) = 24.00±2.65 Humidity (%) = 59.00±21.70 Barometric pressure (hPa) = 1016.00±1.00 Visibility (km) = 11.67±2.08 Wind speed (km·h^-1^) = 11.00±1.73
	2014	N = 44 Age (years) = 25.05±2.02 Body mass (kg) = 79.98±7.34 Height (cm) = 179.89±4.88 BMI (kg·m^-2^) = 24.68±1.57	Temperature (°C) = 24.67±1.53 Humidity (%) = 64.00±5.29 Barometric pressure (hPa) = 1014.67±0.58 Visibility (km) = 11.33±0.58 Wind speed (km·h^-1^) = 10.33±1.15
Autumn N = 145	2013	N = 31 Age (years) = 23.29±3.32 Body mass (kg) = 74.94±5.90 Height (cm) = 178.06±5.33 BMI (kg·m^-2^) = 23.68±2.14	Temperature (°C) = 20.33±4.62 Humidity (%) = 74.33±6.11 Barometric pressure (hPa) = 1015.67±1.53 Visibility (km) = 9.67±0.58 Wind speed (km·h^-1^) = 10.67±3.79
	2014	N = 114 Age (years) = 23.57±3.39 Body mass (kg) = 75.15±5.76 Height (cm) = 177.87±5.23 BMI (kg·m^-2^) = 23.80±2.10	Temperature (°C) = 21.67±3.51 Humidity (%) = 69.33±10.50 Barometric pressure (hPa) = 1015.33±1.53 Visibility (km) = 10.33±0.58 Wind speed (km·h^-1^) = 11.33±0.58
Winter N = 52	2011	N = 35 Age (years) = 23.29±2.07 Body mass (kg) = 75.93±4.94 Height (cm) = 177.67±5.86 BMI (kg·m^-2^) = 24.13±2.25	Temperature (°C) = 12.00±1.00 Humidity (%) = 72.67±6.81 Barometric pressure (hPa) = 1016.00±2.65 Visibility (km) = 8.67±0.75 Wind speed (km·h^-1^) = 13.67±2.31
	2015	N = 17 Age (years) = 23.35±1.84 Body mass (kg) = 77.35±2.37 Height (cm) = 176.59±6.22 BMI (kg·m^-2^) = 24.88±1.67	Temperature (°C) = 11.33±1.53 Humidity (%) = 59.00±7.07 Barometric pressure (hPa) = 1018.67±1.53 Visibility (km) = 9.00±1.41 Wind speed (km·h^-1^) = 16.67±0.58
Spring N = 145	2007	N = 28 Age (years) = 23.68±2.06 Body mass (kg) = 77.30±11.87 Height (cm) = 177.75±6.56 BMI (kg·m^-2^) = 24.43±3.17	Temperature (°C) = 16.67±2.52 Humidity (%) = 64.33±19.35 Barometric pressure (hPa) = 1016.67±2.52 Visibility (km) = 9.50±0.44 Wind speed (km·h^-1^) = 13.00±1.00
	2010	N = 16 Age (years) = 25.56±2.06 Body mass (kg) = 76.31±10.16 Height (cm) = 177.88±4.67 BMI (kg·m^-2^) = 24.08±2.75	Temperature (°C) = 16.33±3.06 Humidity (%) = 69.00±6.56 Barometric pressure (hPa) = 1016.33±6.03 Visibility (km) = 10.00±1.00 Wind speed (km·h^-1^) = 12.33±1.53
	2013	N = 21 Age (years) = 24.81±1.97 Body mass (kg) = 73.60±7.64 Height (cm) = 177.90±5.84 BMI (kg·m^-2^) = 23.27±2.47	Temperature (°C) = 16.00±2.00 Humidity (%) = 55.33±12.06 Barometric pressure (hPa) = 1012.33±2.89 Visibility (km) = 10.33±0.58 Wind speed (km·h^-1^) = 15.00±1.73
	2014	N = 47 Age (years) = 25.26±3.01 Body mass (kg) = 75.37±9.87 Height (cm) = 178.55±5.14 BMI (kg·m^-2^) = 23.58±2.32	Temperature (°C) = 15.67±2.52 Humidity (%) = 74.00±15.72 Barometric pressure (hPa) = 1015.67±0.58 Visibility (km) = 10.00±1.00 Wind speed (km·h^-1^) = 14.33±2.08
	2015	N = 33 Age (years) = 23.82±2.26 Body mass (kg) = 78.79±7.49 Height (cm) = 179.09±5.05 BMI (kg·m^-2^) = 24.53±1.65	Temperature (°C) = 16.67±3.06 Humidity (%) = 55.00±20.81 Barometric pressure (hPa) = 1018.00±1.73 Visibility (km) = 10.00±1.00 Wind speed (km·h^-1^) = 15.67±2.52

BMI = body mass index.

### Training program

The group that underwent the training was required to undergo a planned, programmed and standardized program of military training that lasted for 14 weeks, under strictly controlled conditions of life and work. These conditions involved complete isolation from the outside world, the same kind of food, the same terms of accommodation, the same amount of sleep, the same geographic weather conditions (cold, heat, water, rain, wind), and the same amount of the total burden of training and living conditions, regardless of age, fitness condition, previous work experience or any other factors [7]. The process of this training was approximately similar to the ranger training conducted by the United States 75^th^ Ranger Regiment. Participants could leave the program and resign at any time, while any refusal or failure to perform the tasks that were assigned by the instructor would also result in automatic exclusion. Aside from the content updates on technical and tactical courses, the total volume and intensity of the SCTC training is similar for all training-cycles. Moreover, the SCTC training was conducted regardless of weather conditions (Table 2). Furthermore, the fitness program and testing were applied and monitored by 7 experienced instructors, five of them supervised all the training-cycles.

**Table 2:** Global plan performed by commandos over the 14-week training-cycle.

Phases	Tasks	Goals
**- Phase 1:** Basic **- Duration:** 4 weeks	Tests of fitness abilities, exercise, martial arts, physical and combat drills, land navigation, topography, tactical walking, tactical operations, communications, shooting, movements, avoiding the opponent, first aid in battlefield conditions and explosives.	- Assessment of participants’ skills testing their physical and mental endurance. - Fit participants in various activities designed to put them into various forms of physical and psychological stress, that allows them to make important decisions about joining special forces. - Estimation of the resources and the quality of participants through observation of behavior during extreme effort, analyzing the performance and recording data.
**- Phase 2:** Jungle warfare **- Duration:** 5 weeks	Survival, evasion, resistance and escape from the opponent, walking with load on rugged terrain, exercises, climbing and mountaineering techniques, overcoming natural obstacles, the infiltration of the mountainous area, camping, tactical tasks, patrol, assault courses, field firing weapons and grenades, amphibious infiltration, the implementation of tactical tasks in the amphibious environment, navigation on the swamps, planning and ongoing implementation of special operations in the form of situational exercises with the assumption of action in the opponent’s depth and the constant need to covert movement to evade capture.	- Assessment of the ability of participants when carrying out tasks under conditions of continuous conducting of special operations in the opponent’s territory and to test the physical and mental endurance under extreme conditions (e.g., wake, hunger, thirst, fatigue, inhospitable climate). - Assessment of the ability of participant in a mountain and forest environment when carrying out tasks and testing the physical and mental stamina in the mountain and jungle area. - Assessment of the ability of participants in an aqueous environment when carrying out tasks and testing the physical and mental endurance in and on the not deep water. - Assessment of the synthesis and situational use of previously acquired skills and knowledge.
**- Phase 3:** Urban combat **- Duration:** 5 Weeks	Martial arts and unarmed combat training in an urban environment, tactical tasks, shooting battles and grenades, fight indoor and close quarter fighting (houses, boats. plane), planning and ongoing implementation of special operations in the form of situational exercises with the assumption of action in the opponent’s depth and the constant need to covert movement based on surprise, speed and power.	- Assessment of the ability of participants when carrying out tasks under conditions of continuous conducting of special operations in the opponent’s territory and to test the physical and mental endurance under extreme conditions (e.g., wake, hunger, thirst, fatigue, inhospitable climate). - Assessment of the ability of participants when carrying out tasks in the urban environment and testing the physical and mental endurance in conditions of close combat firearms in closed facilities. - Assessment of the synthesis and situational use of previously acquired skills and knowledge.

The basic phase (28 days) was designed to test and develop the basic military skills, physical and mental endurance, stamina and confidence that a soldier is required to possess in order to successfully accomplish combat missions. The next part (35 days) was designed to develop and assess jungle warfare skills under strictly specific and extreme conditions. The last phase (35 days) was designed to develop and assess urban combat skills, again under strictly specific and extreme conditions (Table 2). In average, the total volume of SCTC was divided into 40% for fitness training and 60% dedicated to technical and tactical training (e.g., practice sessions and class room courses), whereas in some tactical courses we integrate physical training and vice versa. The participants underwent20 h [range: 13–24 h] of training per day, while consuming three or fewer meals daily, totaling about 2,000 calories [range: 800–3200 calories], with an average of 4 h [range: 0–10 h] of sleep per day. Sleep deprivation strategy throughout the SCTC is not regular every day, but it was applied for short duration micro-cycles (e.g. less than 6 days). Participants were expected to maintain a high degree of physical readiness, as operational demands can severely degrade performance capabilities [7, 23].

### Testing procedures

Before the beginning of the study, all participants performed one familiarization session in the week before testing. Data were collected before starting the training-cycle and immediately after the end of the 14-week period. This design was respected over the eight years of the study.

Meteorological data (i.e., temperature, humidity, barometric pressure, visibility and wind speed) were continuously collected from the National Institute of Tunisian Meteorology’s database and from the official Weather Underground website (www.wunderground.com)

For each training-cycle, test data were collected at approximately the same time of day (9:00 a.m. to11:00 a.m.), 2h postprandial (standard breakfast: 10 kcal·kg^-1^, 55% carbohydrates, 33% lipids and 12% protein). Participants performed the tests with commandos’ clothing and shoes but without a bulletproof vest (the mass of the equipment was of ~4 kg, excluding the vest, and consistent during test-retest sessions).

For the warm-ups prior to the testing, their volume was adjusted to reflect the weather conditions, but design and intensity were standardized throughout the seasons. Before the test, each participant performed a standardized battery of warm-ups. After each warm-up, the participants recovered for ~5min and then recommenced the tests. Strong verbal encouragement ensured maximal effort throughout all measurement and training sessions.

The tests consisted of body-weight exercises and a long-distance road-running competition. Fitness characteristics were measured with the following tests: 5-km cross-country[24], pull-ups in 70-seconds [25], push-ups in 70-seconds [25] and sit-ups in 70-seconds [3].These tests are often used as baseline physical evaluations for most special forces units around the world. A pilot study was carried out to ensure the between-days reliability and sensitivity of the tests cited above, with 27 participants performing two measurements of each test, with a one-week interval (Table 3).

**Table 3:** Relative and absolute reliability indices and MDC_95_ of the fitness tests (N = 27).

Variable	Mean±SD		ICC _3,1_ (95%CI)	CV%	SEM (%)	SWC (%)	MDC_95_(%)
	Session 1	Session 2					
**Pull-up test** (score)	16.52±3.89	17.00±3.79	0.913 (0.818–0.959)	9.56	0.47 (2.82)	0.75 (4.48)	1.31 (7.82)
**Sit-up test** (score)	48.59±5.67	48.78±6.25	0.853 (0.704–0.930)	6.64	1.24 (2.55)	1.15 (2.36)	3.44 (7.06)
**Push-up test** (score)	47.37±7.11	47.31±6.15	0.907 (0.806–0.956)	6.21	0.90 (1.90)	1.30 (2.74)	2.49 (5.25)
**5 km cross country** (s)	1234.30±83.17	1204.85±105.07	0.750 (0.523–0.878)	5.49	33.49 (2.75)	17.73 (1.45)	92.82 (7.61)

ICC_3,1_ = Intra-class Correlation Coefficient model 3,1; CV = coefficient of variation; SEM = standard error of measurement; SWC = smallest worthwhile change; MDC_95_ = minimal detectable change at 95% confidence interval.

### Statistical analysis

Data analyses were performed using SPSS version 23.0 for Windows (SPSS, Inc. Chicago, IL, USA). Means and standard deviations (SD) were calculated after verifying the normality of distributions using the Shapiro-Wilk procedure. Training-related effects were assessed by two-way analyses of variance with repeated measures (season×time: [4×2]). To help protect against type II errors, power and effect size were estimated using *ώ* and *ƞ*^*2*^_*p*_, respectively. Bonferroni-adjusted pairwise post-hoc comparisons were performed where appropriate. Effect sizes for pairwise comparisons were calculated as Cohen’s *d*, while comparisons between the percentage of changes in the group was performed by one-way ANOVA. Tukey-adjusted post-hoc comparisons were performed where appropriate, with a paired Student t-test used to compare test-retest performances for the reliability pilot study. The significance for all the statistical tests was accepted at *p*<0.05.

## Results

### Changes in environmental conditions

The means of morning temperatures were significantly (*p*<0.001) higher in summer (26.6±2.3°C) compared with other seasons. Relative humidity (RH) was greater (*p*<0.01) in cool weather (i.e., winter: 65.8±6.9% and autumn: 71.3±8.3%) than during summer (63.4±13.3%) or spring (63.5±14.9%). The comparison of barometric pressure in-between seasons revealed no significant difference (*p* = 0.491). Finally, average wind speed was significantly higher during winter than spring (*p* = 0.39), while the latter was higher (*p*<0.001) than that for summer and autumn.

### Training and season effect

Data were all normally distributed (Shapiro-Wilk: *p*>0.05). For all tests and body mass, there was a significant effect of time and season (*p*: 0.008–0.001; *ƞ*^2^_p_: 0.03–0.84; *ώ*: 0.84–1.00) (Table 4). There were also significant interactions [time×season] for all tests and body mass (*p*<0.001; *ƞ*^2^_p_:0.08–0.43; *ώ* = 1.00) (Table 4). Bonferroni-adjusted pairwise post-hoc comparisons revealed that the performance of physical tests was significantly higher after the training program (*p*<0.05) (Table 4). The post-hoc comparisons in-between seasons at P1 and P2 are illustrated in Table 4.

**Table 4:** Comparison of body mass, pull-up test, sit-up test, push-up test and 5 km cross country test performances, between seasons training commandos groups before and after 14-week trials.

		Summer (n = 124)	Autumn (n = 145)	Winter (n = 52)	Spring (n = 145)	Main Effects		Interactions
						Time	Season	Time×Season
**Body mass (kg)**	**P1**	79.12±7.30†‡	75.11±5.77‡	76.40±4.30‡	76.37±9.58‡	*F* = 2374.57 *p*<0.001 *ƞ*^2^_p_ = 0.84 *ώ* = 1.00	*F* = 3.99 *p* = 0.008 *ƞ*^2^_p_ = 0.03 *ώ* = 0.84	F = 117.62 p<0.001 *ƞ*^2^_p_ = 0.43 *ώ* = 1.00
	**P2**	74.10±6.50	72.18±5.17	74.52±4.28	73.76±8.72			
**Pull-up test (score)**	**P1**	11.38±4.68‡	14.81±4.78¶‡	9.17±3.42¶‡	11.92±4.65‡	*F* = 646.55 *p*<0.001 *ƞ*^2^_p_ = 0.58 *ώ* = 1.00	*F* = 9.99 *p*<0.001 *ƞ*^2^_p_ = 0.06 *ώ* = 0.99	*F* = 38.90 *p*<0.001 *ƞ*^2^_p_ = 0.20 *ώ* = 1.00
	**P2**	15.95±5.13	16.74±5.60	15.31±5.60	18.23±4.97*			
**Sit-up test (score)**	**P1**	43.76±6.96‡	49.86±6.12¶‡	34.67±7.38¶‡	43.53±7.23‡	*F* = 628.13 *p*<0.001 *ƞ*^2^_p_ = 0.58 *ώ* = 1.00	*F = 36*.*42* *p*<0.001 *ƞ*^2^_p_ = 0.19 *ώ* = 1.00	*F* = 62.31 *p*<0.001 *ƞ*^2^_p_ = 0.29 *ώ* = 1.00
	**P2**	50.35±7.36	52.18±6.07§	49.19±6.47	54.98±5.73¶			
**Push-up test (score)**	**P1**	40.19±8.40‡	50.88±7.16¶‡	32.04±8.58¶‡	41.10±9.54‡	*F* = 461.32 *p*<0.001 *ƞ*^2^_p_ = 0.50 *ώ* = 1.00	*F* = 70.53 *p*<0.001 *ƞ*^2^_p_ = 0.31 *ώ* = 1.00	*F* = 55.89 *p*<0.001 *ƞ*^2^_p_ = 0.27 *ώ* = 1.00
	**P2**	52.01±7.83¤	52.96±7.50	39.90±8.76¶	54.71±6.68			
**5 km cross country test (s)**	**P1**	1405.25±222.30¶‡	1239.76±123.78‡	1178.62±100.22‡	1336.88±184.31¶‡	*F* = 289.77 *p*<0.001 *ƞ*^2^_p_ = 0.39 *ώ* = 1.00	*F* = 39.56 *p*<0.001 *ƞ*^2^_p_ = 0.20 *ώ* = 1.00	*F* = 12.64 *p*<0.001 *ƞ*^2^_p_ = 0.08 *ώ* = 1.00
	**P2**	1221.77±117.12¶	1136.24±96.95	1095.55±79.85	1137.07±99.61			

Data are means (±SD), Before training (P1), After training (P2), Effect size (ƞ^2^_p_), Statistical power (ώ).

‡ Significant differences (p<0.001) between before and after training.

¶ Significantly different (p<0.05) from all other seasons.

† Significantly different (p<0.05) from Autumn and Spring.

* Significantly different (*p*<0.05) from Summer and Winter.

§ Significantly different (*p*<0.05) from Winter.

¤ Significantly different (*p*<0.05) from Spring.

**Fig 1:**
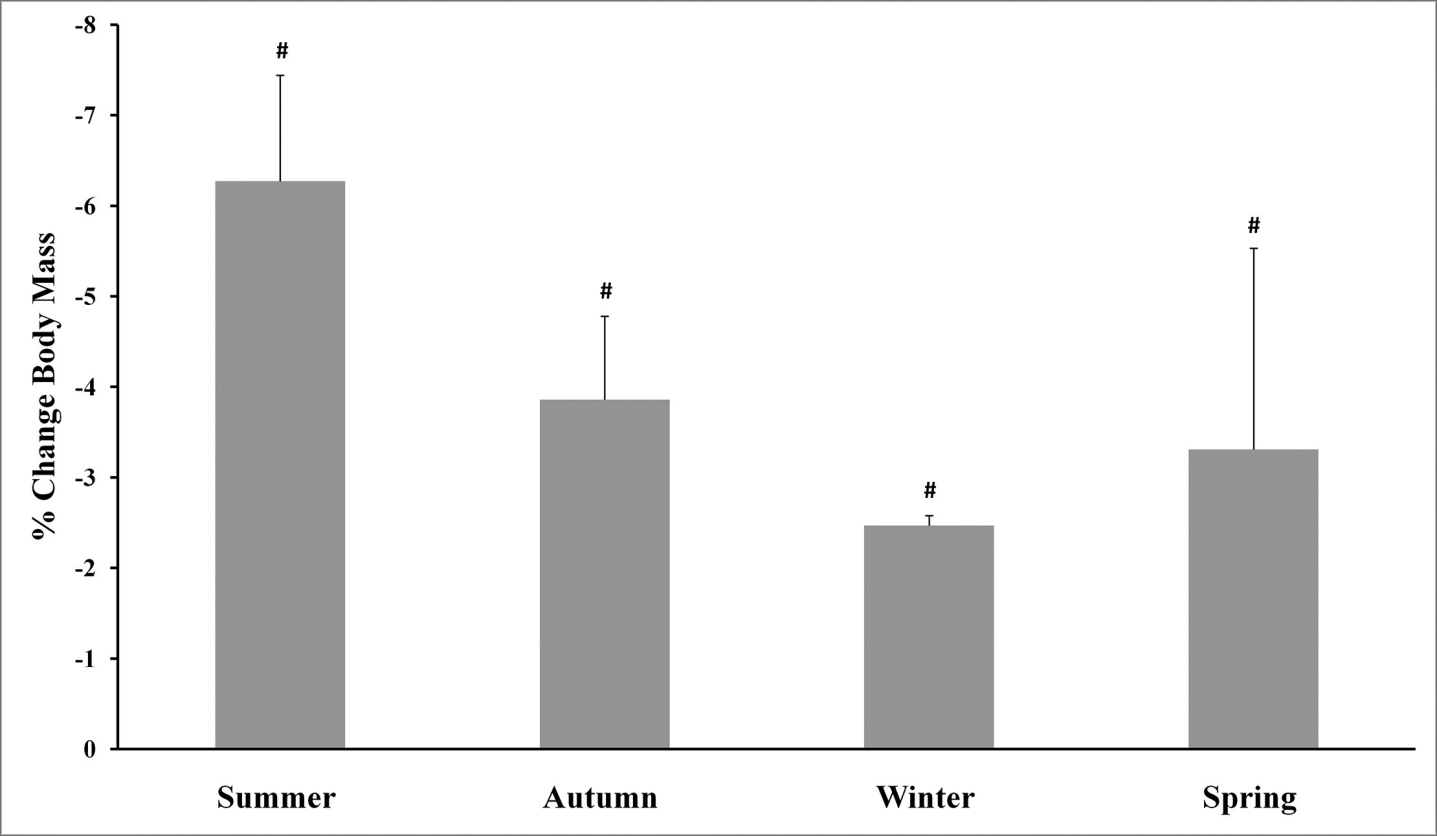
Training associated changes in body mass assessed through seasons over the experimental period. Note: # Significantly different (p<0.01) from all other seasons.

**Fig 2:**
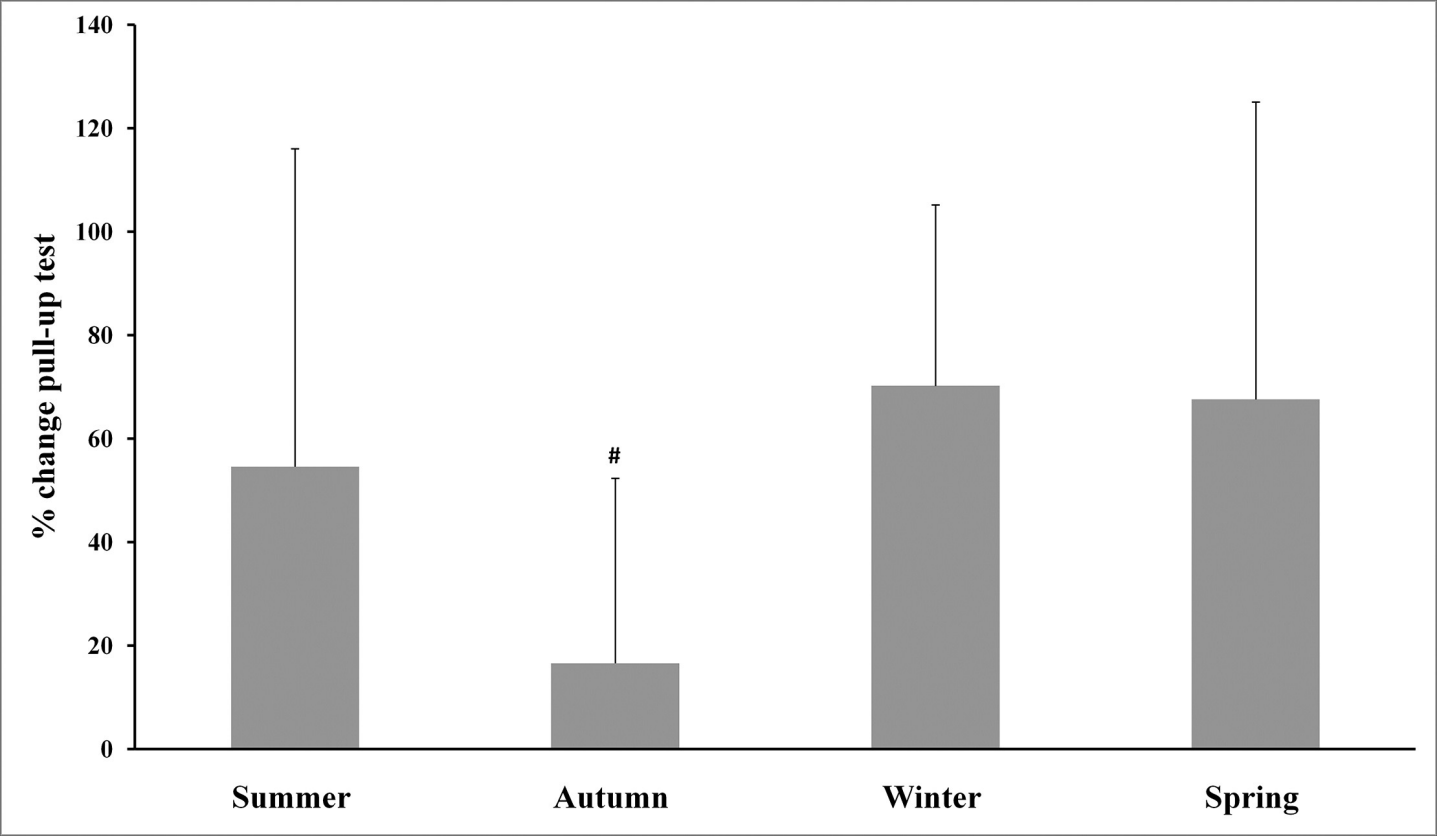
Training associated changes in pull-up test performance assessed through seasons over the experimental period. Note: # Significantly different (p<0.01) from all other seasons.

**Fig 3:**
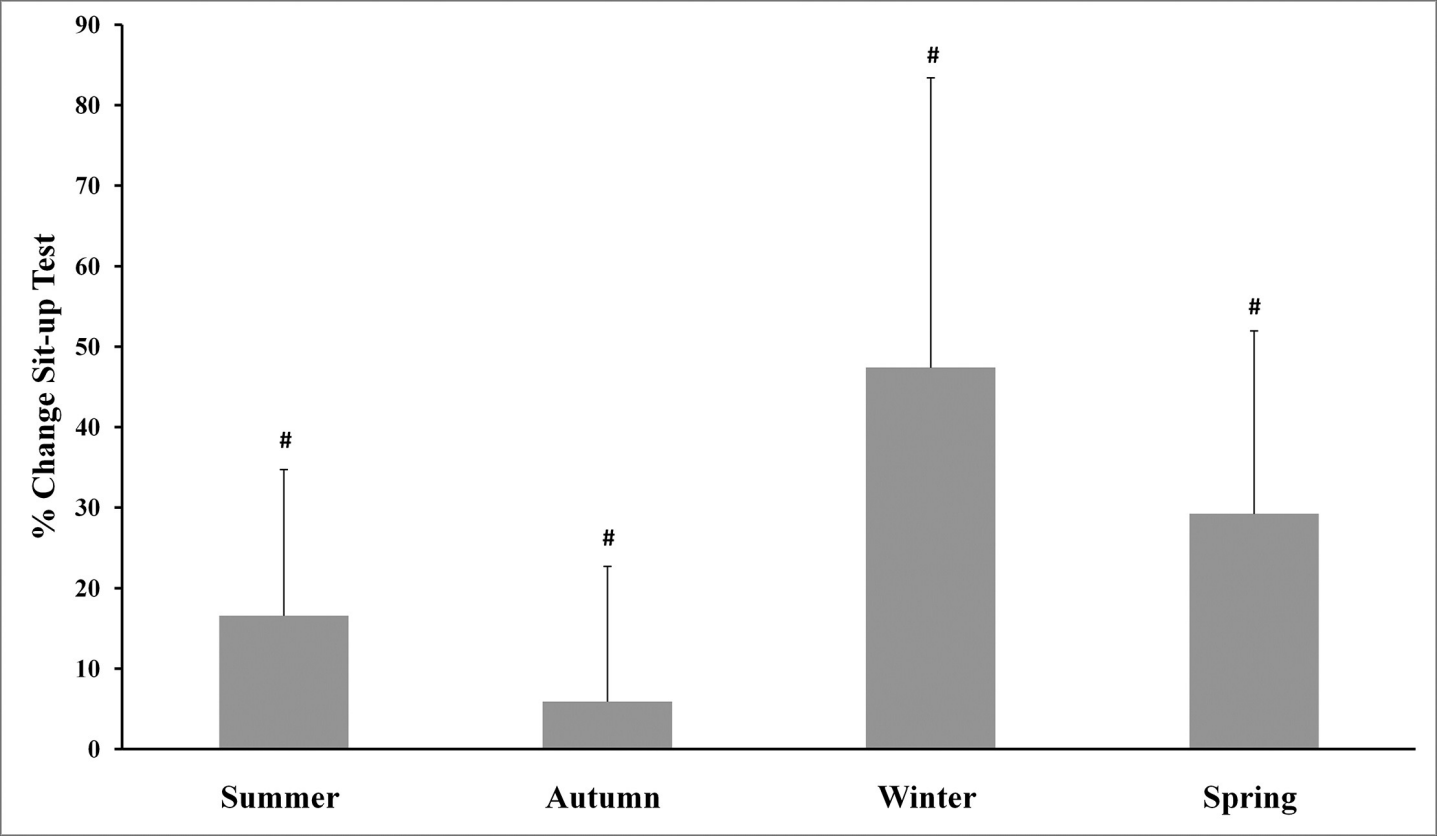
Training associated changes in sit-up test performance assessed through seasons over the experimental period. Note: # Significantly different (p<0.01) from all other seasons.

**Fig 4:**
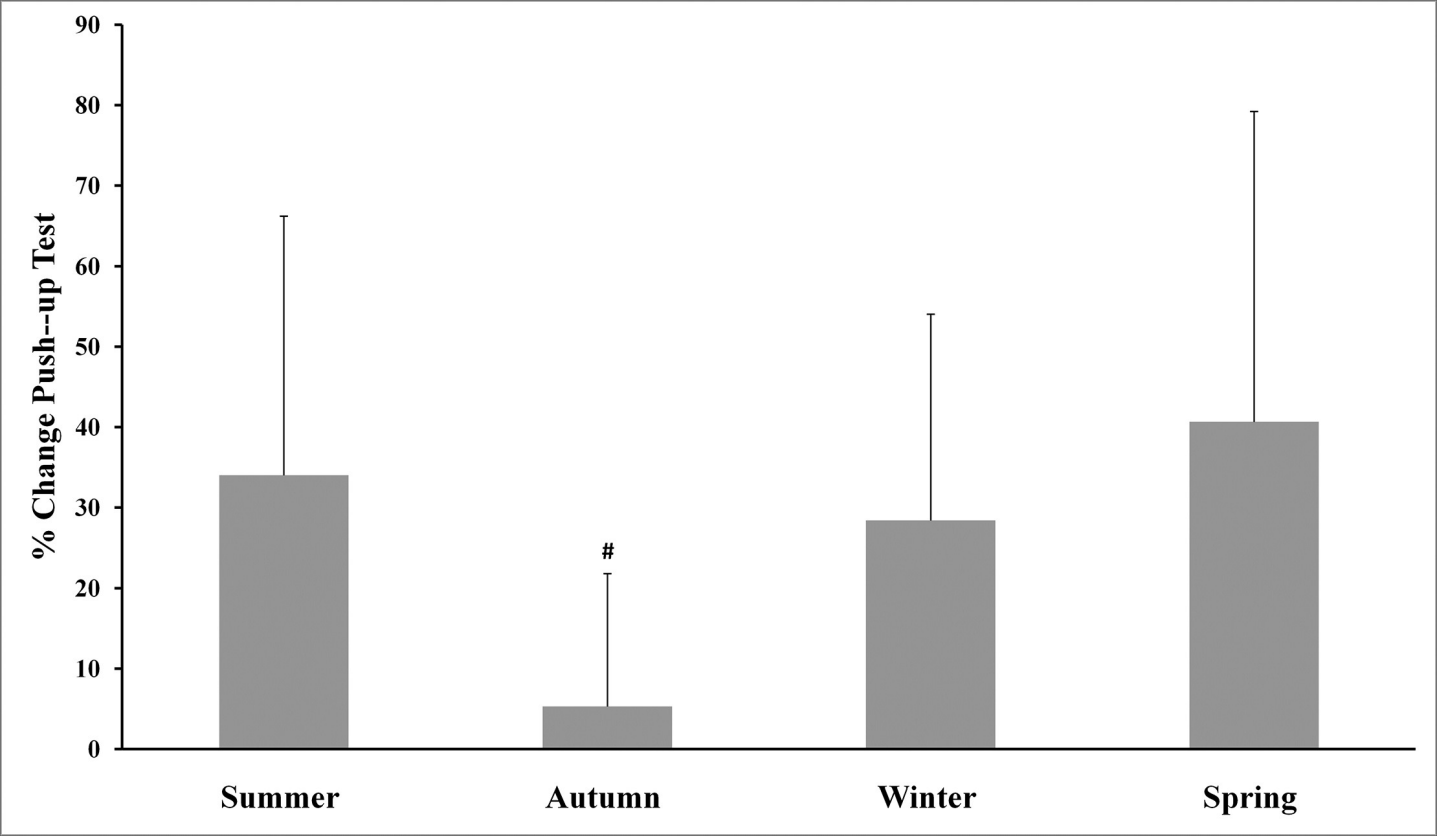
Training associated changes in push-up test performance assessed through seasons over the experimental period. Note: # Significantly different (p<0.01) from all other seasons.

**Fig 5:**
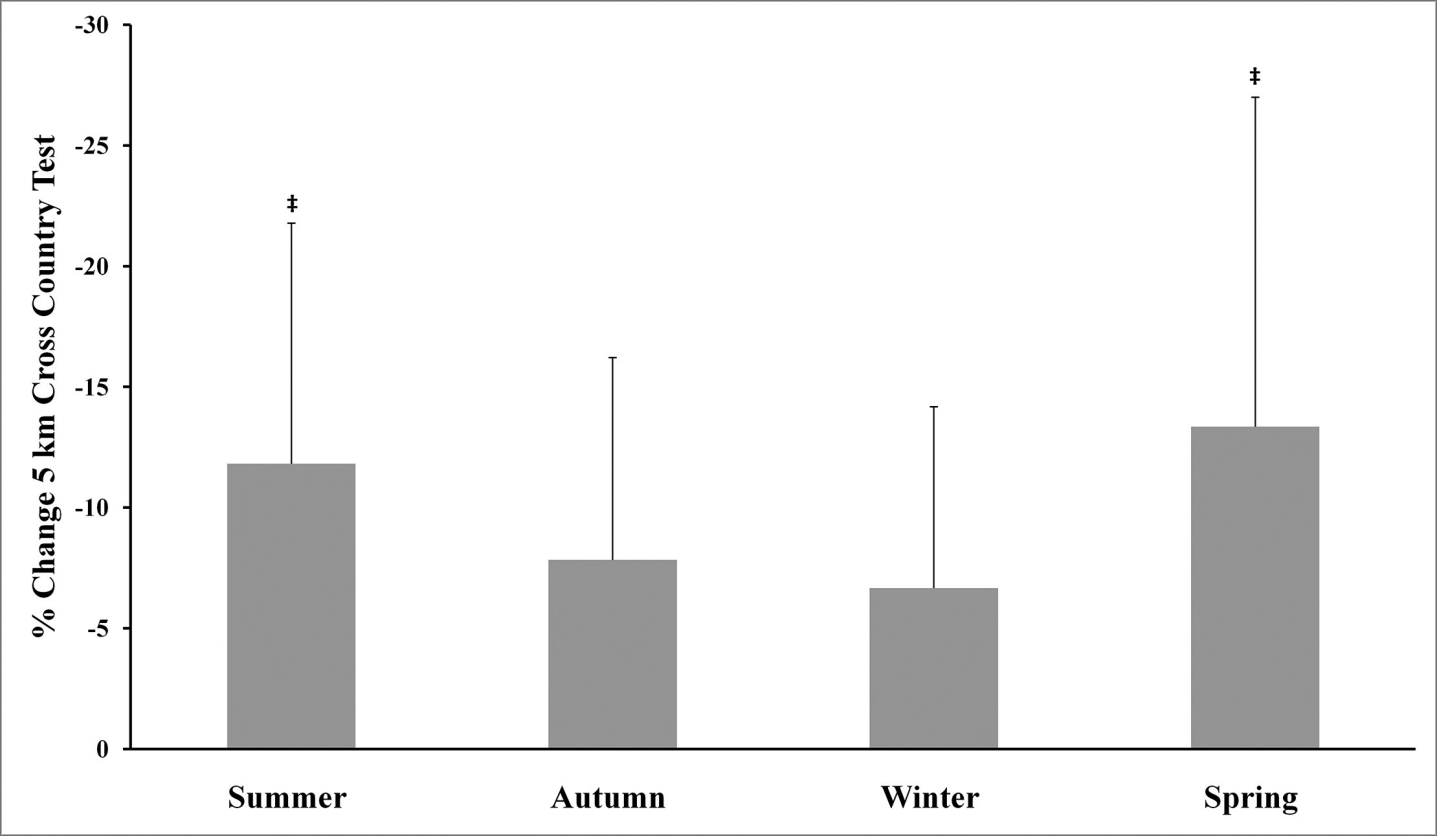
Training associated changes in 5 km cross-country test performance assessed through seasons over the experimental period. Note: **‡** Significantly different (p<0.05) from Autumn and Winter.

The main effects analysis revealed the existence of significant differences between all percentage of changes for body mass and the four physical tests used. The Tukey-adjusted post-hoc comparisons (Figs 1–5) showed that the percentage of changes in the pull-up, sit-up and push-up tests were lower in autumn when compared with other seasons.

## Discussion

The aim of the present study was to investigate the effect of an SCTC program on upper-body strength resistance(push-ups, sit-ups and pull-ups) and running endurance performance (5-km cross-country run), and to determine whether SCTC outcomes could be affected by seasonal variations. The primary finding was that the 14-week SCTC improved upper-body strength resistance and running endurance performance in the trained commandos, independent of the time of year in which the program was held. In addition, seasons significantly affected the percentage of changes in body mass and the performance of all tests. With regard to these percentage changes, improvements in the pull-up, sit-up and push-up tests were lower in autumn compared with other seasons, while improvements in the 5-km cross-country run were higher in spring compared to winter and autumn. The decrease in body mass was greater during summer, compared with autumn and winter.

On the other hand, we demonstrated that the SCTC program had a significant effect on physical performance and body mass. Body mass decreased significantly for all participants (-3.98±1.11%). Similar results have also been reported in several studies using combined endurance and strength training [26], or sprint and resistance training [2, 4]. Emergent evidence confirms that greater improvements in body composition follow resistance training in military solders [11]. Shippee et al. [27] stated that, during the United States 75^th^ Ranger Regiment program, body mass decreased by 16%, which was reflected in the changes in body composition, whereby body fat was reduced by 10%. The average energy deficit during the 14 weeks of training was around 35% and the average amount of sleep was only 4 out of 24 h. Some studies have confirmed that intensive military training has an impact on the reduction of body mass and the percentage of fat [7, 28], which is consistent with the results obtained in this study. Given that the special forces training is characterized by deliberately reduced food intake, in order to create additional stress [29], the results obtained in our study were rather as expected.

Improvements in muscle resistance and endurance capacity, following combined endurance and strength training [11], sprint and resistance training [2, 4], and endurance and track sprint training [30], have been well described in the literature. However, specific training using multicomponent modes (i.e., explosive, resistance and endurance exercises) with specific periodization is less debated in relation to SFSs[11].Body-weight exercises, such as push-ups and sit-ups, are known to stimulate large muscle groups, which require higher blood volume, higher oxygen consumption and more flexibility [31], thus suggesting that an SCTC format is the best alternative to improving cardiovascular and motor performances. In addition, pull-ups and push-ups, as indicators for strength endurance, represent fitness tests that check the preparedness of many armies around the world [7]. There are many specific military activities in which success depends on the skills that can describe this variable (among 20 missions conducted by special forces, nine of them involved the most-valued segments, which involved lifting, pulling, carrying and climbing [32]).

It is noteworthy that our findings indicate that physical performance was significantly different between seasons. It has been well demonstrated that a training-induced increase in performance is more dependent on the balance between environmental temperature and core body temperature, humidity and radiant changes over time [33]. Limiting evaporation, due to being faced with metabolic heat, ambient heat and increased sweating in a humid environment, commonly contributes to a decrease in the physiological ability needed to produce a maximum force in comparison with “so-called” normal conditions at ambient temperature or thermal neutrality [33]. In North Africa, the moderately warm season begins in mid-April and lasts until mid-June, before temperatures begin to rise to >28°C.On account of this geographical feature, the region experiences rainy winters and hot, dry summers. In the current study, moderately warm temperatures were registered during mid-spring and the beginning of the summer season (temperature: ~26°C and humidity: ~62%). The cross-country test performed in this period of the year was characterized by elevated performance following the training of all participants. Such a result appears to be related to the specific environmental conditions, including a low humidity rate and a moderately hot temperature [33]. It is well known that high humidity is a limiting factor in the sweating process by “hidromeiosis”, especially during the prolonged exertion of effort [33]. In addition, exercise under heat conditions is usually accompanied by severe changes in fluid balance and a high magnitude in the metabolic rate, which can induce early fatigue and disturbances in cardiorespiratory responses during a prolonged workout [33]. When the human body reaches an elevated internal temperature (between 39°C and 40°C), the nervous system diminishes its ability to recruit muscle fibers, alters stress hormones and induces greater acidosis, which alters muscle function [34]. The moderate temperature with a low humidity rate during the spring season appears to offer adequate conditions for endurance capacity training and endurance competition events.

Furthermore, we only observed an important significant increase in push-up, pull-up and sit-up performance during cool seasons (autumn and spring) compared to the mid-warm season. The percentage of improvements in the pull-up, sit-up and push-up tests was lower in autumn, compared with other seasons, while the percentage of change in the 5-km cross-country test was higher in spring compared to winter and autumn. Only a few studies have reported similar data (percentage of changes) in response to strenuous workouts [35], while most other studies have measured this percentage of change using performance indices [36]. It should be noted that small training alterations, including dietary adjustments, can be influenced by weather fluctuations. Further research is needed to better understand the acclimation process among commandos and help interpret the results of the current study. Improvements in neuromuscular characteristics will not be possible without a reduction in the amount of military training based on endurance, as well as an increase in training intensity for power and strength during military training [7]. Montain and Young [37] have suggested that such an operational tempo was not recommended for more than 10 continuous days, whereas, in this study, a period of 10 weeks (phases 2 and 3) was involved. To realize optimum physical performance improvements, warfighters should place special emphasis on achieving high levels of strength, power and muscularity through optimal physical training programs and food [7].Importantly, the used physical fitness tests in this study, are body weight-bearing exercises (i.e., pull-ups, push-ups and sit-ups in 70-seconds) or body weight dependant (i.e., 5 km cross-country run). In addition to the improvement in physical fitness tests performances, this study also showed a decrease in body mass of participants. Therefore, improvements in tests performance cannot be attributed solely to the effect of training but also at least partly to the decrease in body mass of participants.

Other studies have examined the effects of SCTCs[11], but this is the first study to follow-up the gains in muscle performance over the seasons. There were several limitations to the study in addition to those already mentioned. First, without a control group, we cannot know whether the planned training protocol alone could have potentially caused the positive effects. Only selected physiological characteristics were analyzed in this research: upper-body strength resistance and running endurance performance. It would be beneficial for future research to investigate commando training and seasonal effects on other capacities, which are key to the physical requirements of SFSs.

As recommendation, the results of the present study should be of interest to coaches and other experts working with SFSs. This study has demonstrated that the SCTC program in question may be effective in improving the strength resistance of upper-body muscle and running endurance performance. In addition, as SCTC programs require individualization and periodization as much as possible, based on the planned special forces tasks, as well as the physical fitness and body-mass index of soldiers, they should take into account the relevant environmental conditions if optimal performance and training effects are to be achieved. Indeed, the studied SCTC program resulted in more efficient upper-body strength resistance during winter and spring, and greater running endurance performance during spring and summer. Furthermore, the best performance took place during autumn and spring for strength resistance exercises, and during winter for running endurance efforts. Our findings should help fitness coaches to develop their acclimatization (long-term) and cooling/warm (short-term) strategies in order to optimize the performance and efficiency of their training programs. It would also be appropriate to develop nutritional strategies for greater control over body-mass changes during summer training.

## Conclusion

In summary, the 14-week SCTC program resulted in increased upper-body strength resistance and running endurance performance in commandos. Although the best cross-country running performances took place in winter, the improvement rate was always greater during spring and summer. For upper-body strength resistance (e.g., pull-ups, push-ups and sit-ups), performance improvements were greater during cool weather (autumn and spring), while improvements after the SCTC program were greater during winter and spring than other seasons. Further research is needed to understand the factors that result in poorer endurance performance during summer and strength resistance efforts during winter.
